# Contextual Updating in Attentional Orienting Relies on the Right Temporoparietal Junction: Evidence From rTMS

**DOI:** 10.1111/ejn.70429

**Published:** 2026-02-20

**Authors:** Giorgia Parisi, Chiara Mazzi, Elisabetta Colombari, Sonia Mele, Silvia Savazzi

**Affiliations:** ^1^ Perception and Awareness (PandA) Laboratory, Department of Neuroscience, Biomedicine and Movement Sciences University of Verona Verona Italy

**Keywords:** attentional reorienting, contextual updating, location‐cueing paradigm, repetitive transcranial magnetic stimulation, right temporoparietal junction

## Abstract

The right temporoparietal junction (rTPJ) has been associated with multiple cognitive functions. Particularly, its involvement in attentional processes has been proposed, representing a key node in shifting and reorienting visuospatial attention toward unexpected stimuli. However, more recent evidence has demonstrated a more postperceptual function, suggesting a crucial engagement of rTPJ in contextual updating mechanisms. Therefore, considering the lack of consensus, the aim of the current study was to elucidate rTPJ contribution to attentional processes by applying repetitive transcranial magnetic stimulation (rTMS) throughout the administration of a location‐cueing paradigm. Importantly, the latter was built in order to permit the disentangling of two critical attentional mechanisms: visuospatial reorienting and contextual updating. Data were collected from young healthy participants performing a discrimination task in a Posner‐like paradigm while online rTMS interfered with rTPJ activity starting from 250 ms after the target onset (three pulses at 20 Hz per trial). Comparing behavioral outcomes of the active rTMS condition with a Sham condition (i.e., no stimulation), allowed us to directly observe rTMS effects on the neural processes at hand. Our findings showed an intact advantage of being attentionally focused on the attended location along with the cost of rearranging attentional resources to the unattended location in both rTMS and Sham condition, thus supporting that rTPJ should not be involved in triggering the reorienting of attention toward unexpected locations. Rather, rTMS selectively affected participants' ability to update the predictive attentional context, letting us conclude that rTPJ could be engaged in postperceptual and contextual updating mechanisms.

## Graphical abstract

The right temporoparietal junction (rTPJ) has been proposed to support both attentional reorienting and contextual updating. Using online rTMS during a location‐cueing task, we dissociated these mechanisms and tested rTPJ involvement. rTPJ disruption was found to selectively impair contextual updating without affecting attentional reorienting, highlighting a postperceptual role.

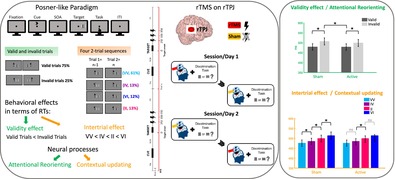

**Abbreviations**
CV: cue validity
DAN: dorsal attention network
FDI: first dorsal interosseous
fMRI: functional magnetic resonance imaging
II: invalid trial‐invalid trial
IT: inferior temporal cortices
IV: invalid trial‐valid trial
MRI: magnetic resonance imaging
PPC: posterior parietal regions
RT: reaction time
rTMS: repetitive transcranial magnetic stimulation
rTPJ: right temporoparietal junction
SDs: standard deviations
SEM: standard error of the mean
TMS: transcranial magnetic stimulation
VAN: ventral attentional network
VI: valid trial‐invalid trial
VV: valid trial‐valid trial

## 1 Introduction

The right temporoparietal junction (rTPJ) is a human brain region comprising the ventral nodes of the right parietal cortex, namely, the supramarginal and angular gyri, together with the right caudal portion of the superior temporal gyrus contiguous to the posterior ending of the Sylvian fissure (Decety and Lamm 2007; Doricchi et al. 2022; Patel et al. 2019). In the last two decades, much effort has been made to identify the exact nature of its role in supporting human cognition, though without attaining a collectively shared interpretation. This issue has been prompted by evidence suggesting both the involvement of rTPJ in multiple cognitive processes and its parcellation in anatomically different subregions (Caspers et al. 2006). Indeed, it is still unclear whether these subsectors mediate distinct domain‐specific functions, as stated by the “Fractionation view” (Krall et al. 2015; Nelson et al. 2012; Scholz et al. 2009), which associates distinguishable rTPJ subregions with specific cognitive functions, or, alternatively, rTPJ underpins one common cognitive mechanism across distinct cognitive domains (Doricchi et al. 2022). The latter perspective, known as the “Overarching view” (Cabeza et al. 2012; Schuwerk et al. 2017, 2021), suggests a unifying overarching role of rTPJ in monitoring surrounding contingencies. More specifically, this brain region has been linked to the more general function of “Contextual Updating” (Geng and Vossel 2013), which refers to the ability to update internal models of the current behavioral context to build expectations and responses accurately.

Despite the still‐existing debate, the rTPJ engagement in the visuospatial attention domain is widely accepted (Dugué et al. 2018), recognizing it as a critical hub of the ventral attentional network (VAN), whose activity is, in turn, associated with endogenous reorienting of attention toward unexpected but behaviorally relevant visual stimuli (Corbetta et al. 2008). According to this model, rTPJ was initially thought to contribute to the reorienting process by interrupting the top‐down deployment of attention performed by dorsoparietal regions (i.e., the cortical regions included in the dorsal attention network, [DAN]) and successively allowing the same cortical nodes to proceed with reorienting attention toward unexpected but task‐relevant stimuli (Corbetta and Shulman 2002). Importantly, this perspective would entail an early recruitment of rTPJ during visuospatial attentional processes. However, recent findings have provided evidence for the opposite scenario, revealing a later attentional engagement of rTPJ occurring after 300 ms from the target onset onward (Mengotti et al. 2017; Parisi et al. 2020). This evidence is in line with the association between rTPJ and the P3b component (Polich 2007) which, indeed, represents a sub‐component of the electrophysiological potential P300, usually occurring in a relatively late post‐target time window (300–500 ms). Importantly, P3b activity has been shown to emerge from a distributed cortical network, with major contributions from posterior parietal regions (e.g., PPC) and inferior temporal cortices (e.g., IT), in addition to temporoparietal areas such as rTPJ (Bledowski et al. 2004). Moreover, P3b is traditionally considered a neurophysiological correlate of contextual updating (Arjona and Gómez 2013; Donchin and Coles 1998; Polich 2007), which, in the visuospatial attentional framework, should arise after endogenously reorienting attention toward unattended target locations (Mengotti et al. 2017). Accordingly, rTPJ would contribute to visuospatial attentional processes by updating internal models of the attentional context in order to create expectations and properly guide future actions (Doricchi et al. 2010; Parisi et al. 2020). It, thus, appears evident that there is no unequivocal consensus, even concerning the contribution of rTPJ to the attentional sphere, remaining unclear whether it has a responsibility in subserving visuospatial endogenous reorienting or a more comprehensive role of contextual updating.

The purpose of the current study is to elucidate the role of rTPJ in visuospatial attentional processes by employing a behavioral task where attentional reorienting and contextual updating can be disentangled and separately investigated. A modified version of the classical location‐cueing paradigm (Posner 1980) can address this issue. The visuospatial adaptation of this paradigm is usually characterized by a central predictive cue that may (valid trial) or may not (invalid trial) predict the location of an impending visual target (Capotosto et al. 2012; Natale et al. 2009; Vossel et al. 2006). Reaction times (RTs) related to valid targets are typically faster than those related to invalid targets, reflecting the so‐called “validity effect.” This effect results from the advantage of being attentionally oriented to the cued location and the cost produced by redirecting attentional resources from the location indicated by the cue to the uncued one (Posner 1980). Furthermore, it has been shown that participants performing location‐cueing paradigms execute a cognitive assessment concerning the validity/invalidity of a trial, leading to behavioral effects that carry over to the subsequent trial (Gómez et al. 2009). These consist of greater RT advantages for valid trials and greater RT costs for invalid trials following valid, rather than invalid, trials (Arjona and Gómez 2011). These behavioral mechanisms describe the so‐called “intertrial (Validity/Invalidity) effect”, which explains the established idea that attentional processes are critically affected by trial history and the current probabilistic context (Vossel et al. 2015).

Examining the validity effect and the intertrial effect provides the possibility to segregate the endogenous reorienting and the contextual updating, respectively: The validity effect stands for a clear outcome of endogenously reorienting attention toward the unexpected but behaviorally relevant target location, while the intertrial effect reflects the influence that the assessment of the validity condition in one particular trial (*n*‐1) has on the performance of the following trial (*n*). Such trial‐by‐trial adjustments are widely considered a hallmark of contextual updating, as they reflect the continuous integration of recently processed information into the internal model that rules expectations about forthcoming events. Crucially, the intertrial validity effect captures the extent to which participants incorporate the outcome of a given trial (i.e., whether the cue was informative or misleading) to refine their anticipatory set for the next one. This mechanism aligns with classical frameworks of contextual updating, which posit that the cognitive system constantly recalibrates its predictions about environmental contingencies to optimize performance (Donchin and Coles 1998; Polich 2007). Accordingly, the intertrial effect provides a behavioral index of how efficiently individuals revise and adjust their probabilistic beliefs about cue‐target relationships on an ongoing basis, thus offering a reliable measure of contextual updating processes (Arjona Valladares et al. 2017; Vossel et al. 2015). Hence, in order to shed light on the precise role of rTPJ in visuospatial attentional processes, we employed a noninvasive neurostimulation technique, i.e., the transcranial magnetic stimulation (TMS), during the administration of a location‐cueing paradigm. It is well known that online TMS (i.e., administered simultaneously with the execution of the behavioral task) interferes with the neural activity of a definite cortical region with a spatial resolution of the order of millimeters (Schuwerk et al. 2021) and good temporal precision. Applying TMS over rTPJ within a specific time window enabled us to directly assess the involvement of rTPJ in visuospatial attentional dynamics: Any effect on the participants' task performance resulting from the interference of TMS would be informative on the rTPJ's role in attentional reorienting and contextual updating.

## 2 Materials and Methods

### 2.1 Participants

Twenty‐four right‐handed (as assessed by the Edinburgh Handedness Inventory, Oldfield, 1971) adults (four males) were recruited for the study. Their ages ranged between 20 and 30 years (mean age ± standard deviation: 24.1 ± 3.5), and they had normal or corrected‐to‐normal vision. Written informed consent was obtained from each volunteer in accordance with the principles laid down by the Declaration of Helsinki. The experimental protocol has been approved by the local Ethics Committee. As assessed by a safety screening questionnaire (adapted from Rossi et al. 2009), all participants were negative for the risk factors associated with TMS: None reported any history of epilepsy or migraine, cardiac pacemaker, neurological disorders, current treatment with any psychoactive medication, and pregnancy. Eighteen participants received reimbursement for their participation, while six participants were internship students from the University of Verona. All but one (author E.C.) participants were naïve to the aims of the study.

### 2.2 Experimental Procedure

In a within‐subjects design, participants underwent two experimental sessions over 2 days. In each experimental session, both active TMS and sham stimulation were delivered and interspersed by a 20‐min break, independently from the starting stimulation, which was counterbalanced across participants. Moreover, the order of active TMS and sham stimulations was alternated between the two experimental sessions for each participant.

### 2.3 Behavioral Task

Participants were individually tested in a dimly lit testing room. During each experimental session, they sat in front of a 24‐in. LCD monitor (resolution 1920 × 1080, refresh rate of 144 Hz) at a viewing distance of 57 cm. They leaned on a chin rest with forehead support so that their eyes could be adjusted to the center of the screen, and head movements could be restricted when TMS was positioned.

**FIGURE 1:**
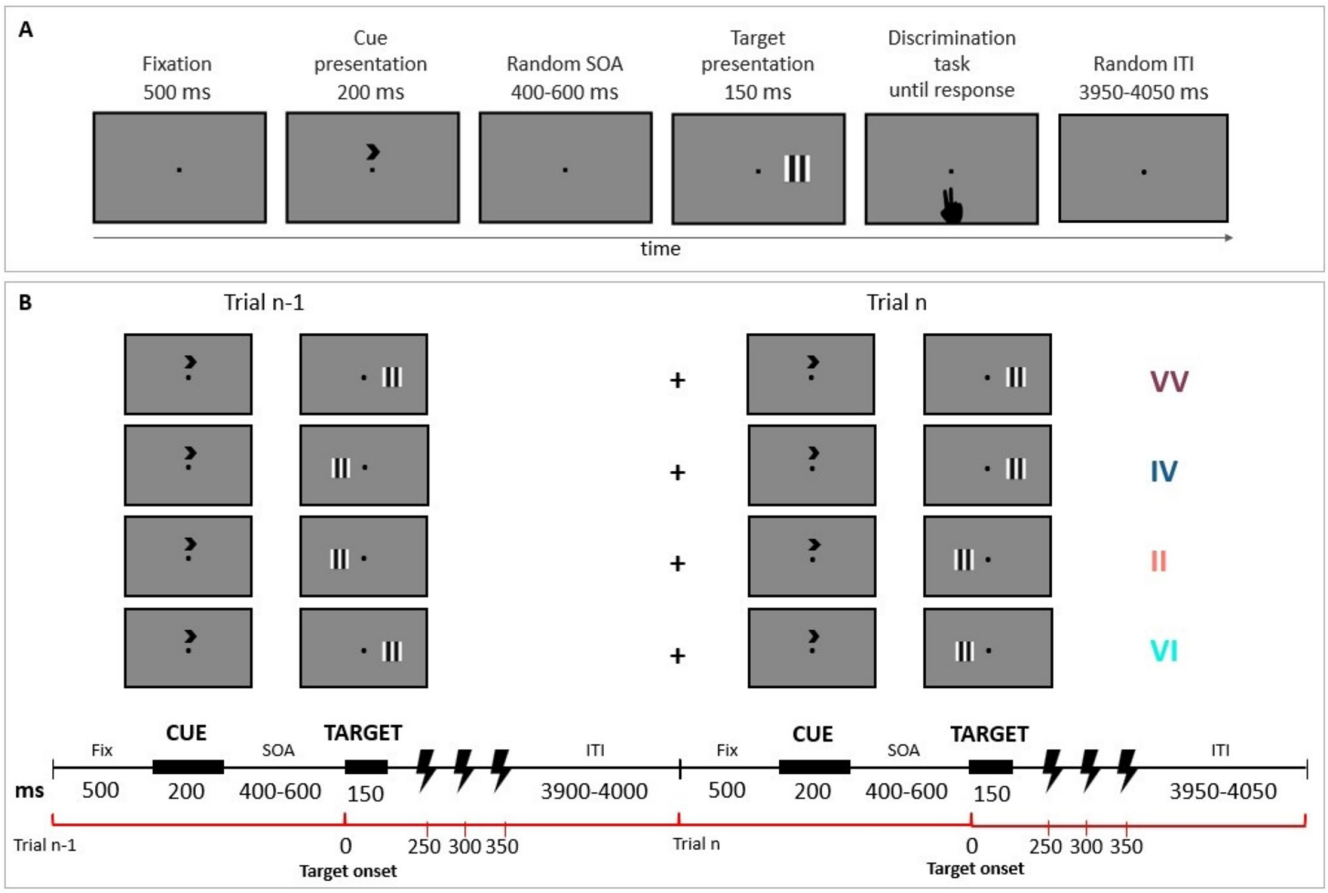
Experimental paradigm. (A) Timeline of the experimental paradigm for a right validly cued trial. A fixation point was presented for 500 ms, followed by a central predictive cue lasting 200 ms. After a randomly jittered interval between 400 and 600 ms, the visual target was presented for 150 ms. Participants had to discriminate the target orientation (i.e., vertically or horizontally oriented gratings). (B) The four types of the considered two‐trial sequences are shown, together with the temporal sequence of events of trial *n*‐1 and trial *n* (reported in the inferior part of the figure). rTMS was delivered in every trial at 20 Hz starting from 250 ms (i.e., 250, 300, and 350 ms) after the target onset.

A location‐cueing paradigm with central cueing was employed (Posner 1980). Stimuli were generated using E‐Prime 2.0 software (E‐Prime Psychology Software Tools Inc., Pittsburgh, PA, USA) and consisted of vertical or horizontal, black‐and‐white, 2° square gratings. Participants were asked to maintain fixation on a centrally presented black point lasting for 500 ms on a gray background. Afterward, a central black predictive cue (i.e., an arrow) was shown above the fixation point (duration 200 ms). After a randomly jittered interval between 400 and 600 ms (randomly jittering the interval between cue and target occurrences reduces temporal expectation consequences, while still providing a time window sufficient for attentional engagement), the target was presented for 150 ms at an eccentricity of 2° from the fixation point to the inner edge along the horizontal meridian. The intertrial interval was random, ranging from 3950 to 4050 ms (Figure 1A). Participants were asked to discriminate the target's orientation as fast as possible by pressing the “b” key with their right index finger or the “n” key with their right middle finger on a standard QWERTY IT keyboard. Instructions were counterbalanced across participants: “b” had to be pressed when vertical targets appeared, “n” had to be pressed when horizontal targets appeared, and vice versa. Horizontal and vertical trials randomly occurred in each block with the same probability. Likewise, left and right targets were equally distributed within a single block, appearing in a random fashion.

Each experimental session was divided into 10 blocks, for a total of 20 blocks per participant. The percentage of cue validity (%CV) was fixed so that in each block, 75% of trials were valid, that is, when the cue gave the correct indication about the impending target location, and 25% of trials were invalid, that is when the target appeared on the unattended side. Importantly, the cue direction was kept constant throughout a block: In half of the blocks (i.e., 10), the cue pointed to the right, while in the other half, it pointed to the left visual hemifield. Participants were informed that the location indicated by the cue had a higher probability of including the target while unaware of the exact CV%. Moreover, four two‐trial sequences were considered in building and programming the task. These sequences were valid trial‐valid trial (VV), invalid trial‐valid trial (IV), invalid trial‐invalid trial (II), and valid trial‐invalid trial (VI) (Figure 1B). All sequences were included in each block in a pseudorandom order, aiming to obtain at least 60 presentations for each sequence within the whole task (see Table 1 for the exact number of presentations of each sequence per block). Each participant was presented with the same sequence of trials in each block, which consisted of 39 valid trials and 13 invalid trials, for a total of 1040 trials (780 valid and 260 invalid) per participant. In each experimental session, active TMS and sham stimulation were administered for five consecutive blocks, respectively, and interspaced with a 20‐min break. A total of 10 active blocks and 10 sham blocks were thus obtained per participant. Accordingly, block order, which was random during the first experimental sessions, remained unchanged for each participant over the second experimental session.

**TABLE 1:** Number of presentations of each sequence per block.

Block\sequence	VV	IV	II	VI
**Block 1**	31	7	7	6
**Block 2**	32	6	7	6
**Block 3**	31	7	6	7
**Block 4**	31	7	6	7
**Block 5**	32	6	7	6
**Block 6**	31	7	7	6
**Block 7**	32	6	7	6
**Block 8**	31	7	6	7
**Block 9**	31	7	6	7
**Block 10**	32	6	7	6

### 2.4 TMS Protocol

Triple‐pulse magnetic stimulation was delivered over rTPJ through a 70‐mm figure‐of‐eight coil connected with a Magstim Rapid2 system (Magstim Company Limited, Whitland, UK). The TMS coil was held tangentially to the scalp, with the handle pointing backward. Three pulses at 20 Hz per trial were administered starting at 250 ms onward after the target onset (Figure 1B). The TMS pulse triggers were computer‐controlled via the same script as the behavioral task.

This repetitive short‐train protocol was intentionally selected because brief high‐frequency rTMS bursts (such as 2–5 pulses at 10–20 Hz) are widely recognized as one of the most reliable and well‐established approaches for inducing transient functional interference (Lega et al. 2019; Sack and Linden 2003; Sandrini et al. 2011). Their repetitive nature allows the stimulation to disrupt neural processing more effectively than a single pulse, while still avoiding longer lasting neuromodulatory effects. Therefore, delivering the triplet shortly after target onset enabled us to interfere with the temporally specific contribution of rTPJ to attentional mechanisms.

Stimulation intensity was set to 100% of each participant's resting motor threshold (rMT) derived from the left primary motor cortex activating the right first dorsal interosseous (FDI) muscle. The rMT was the lowest pulse intensity eliciting five out of 10 motor‐evoked potentials with an amplitude of at least 50 μV recorded through BrainAmp amplifiers (Brain Products GmbH, Munich, Germany—Brain Vision Recorder) and four Ag/AgCl electrodes. Consequently, the mean stimulation intensity across participants was 59% ± 6% (mean ± SD).

A neuronavigation software (SofTaxic, E.M.S., Bologna, Italy) combined with a 3D optical digitizer (Polaris Vicra, NDI, Waterloo, Canada) was used throughout the whole experimental session to guide and constantly monitor the TMS‐coil placement with an accuracy of 2 mm. Moreover, 12 participants out of 24 underwent a T1 magnetic resonance imaging (MRI) scan prior to this experiment, providing the possibility of implementing T1‐weighted images into the neuronavigation system. As a result, rTPJ (i.e., the stimulation site) coordinates were chosen individually for each of these participants by visually checking the anatomical localization of rTPJ (i.e., the posterior part of the right temporal gyrus) in each participant's brain and adjusting them according to the rTPJ coordinates employed by Mengotti et al. (2017). For those participants whose structural MRI was not available, an estimated MR‐based head model was individually created using the neuronavigation software and a warping procedure. This latter was based on four fiducial points (nasion, inion, and preauricular points) along with a large number of scalp points (247 points). A virtual reconstruction of the scalp surface was then generated according to a specific procedure based on scalp point digitization. Subsequently, the reconstruction was used to compute 345 scalp reference points based on the international 10–5 system (a set per participant) through which the averaged standard template MRIs were adjusted. The rTPJ coordinates utilized for the second half of the sample consisted of the mean rTPJ coordinates from the first 12 participants (i.e., *x* = 66, *y* = −41, *z* = 18).

A 70‐mm figure‐of‐eight placebo coil (Magstim Company Limited, Whitland, UK) was employed for the control sham stimulation, appearing identical to the regular coil. The sham coil was held tangentially to the surface of the scalp over rTPJ in order to mimic the placement, the noise, and the mechanical vibration of TMS without actually stimulating the brain tissue. Participants were provided with commercial disposable earplugs to protect them from the machine's noise (Rossi et al. 2009) during both active and sham stimulation and to prevent responses from being influenced by the intensity of the coil click.

### 2.5 Behavioral Data Analysis

Data were processed using E‐Prime 2.0 software (Psychology Software Tools Inc., Pittsburgh, PA, USA) and analyzed with Jamovi (The jamovi project 2022; Jamovi version 2.3, computer software, retrieved from https://www.jamovi.org). For each participant, horizontal and vertical trials were systematically collapsed. Mean reaction times (RTs) and the corresponding standard deviations (SDs) were computed for each stimulation condition (Active and Sham), each validity condition (valid and invalid, independently from cue and target side), and each sequence (VV, IV, II, and VI) across participants. In order to control for unspecific effect and directly investigate and operate on the correct cognitive mechanism, much attention was paid to the intertrial effect at baseline level (i.e., Sham condition). Specifically, the difference between IV and VV and the difference between VI and II were controlled to properly reflect the updating process during the Sham condition: Their outcomes had to be equal to or greater than zero. Accordingly, participants whose differences resulted in being positioned under the 10th percentile were excluded from further analyses, for a total of six participants ruled out. Furthermore, data from one participant were excluded from the analyses due to stimulation issues. Thus, the final sample comprised 17 participants (four males, mean age ± standard deviation: 24 ± 3.2). The adequacy of this sample size was verified by employing G*Power software (v. 3.1.9.7) and assuming a power of 95% and a significance level of 5%, which resulted in a minimum required sample of 15 participants (critical *F* = 4.600; actual power = 0.958). In addition, this sample size is also consistent with that used in a previous work investigating analogous behavioral mechanisms (Gómez et al. 2009).

Trials with RTs exceeding ±3SDs from the mean in each condition were considered outliers and removed from the subsequent analyses. After this procedure, mean values were obtained from an average of 96% and 95% for valid (Sham and Active, respectively) conditions and 93% and 94% for invalid (Sham and Active, respectively) conditions.

Firstly, RTs were entered in a two‐way 2 × 2 repeated‐measures analysis of variance (ANOVA) with stimulation (Active and Sham) and validity conditions (valid and invalid) as within‐subject factors. Furthermore, a second two‐way 2 × 4 repeated‐measures ANOVA with stimulation (Active, Sham) and sequence (VV, IV, II, and VI) conditions as within‐subject factors was conducted by analyzing RTs related to the second trial of each two‐trial sequence. A significance threshold of *p* < 0.05 was set for all statistical analyses. The data are reported as the mean ± standard error of the mean (SEM). Effect sizes were estimated using the partial eta squared measure (*ƞ* ^2^_p_). Where needed, Bonferroni‐corrected post hoc *t*‐tests were applied.

In addition to RT analyses, accuracy was computed for each participant across all experimental conditions. Accuracy was calculated for valid and invalid trials in both stimulation conditions (Active and Sham) and for each of the four sequences (VV, IV, II, and VI). Two repeated‐measures ANOVAs were conducted: a 2 × 2 ANOVA with validity (valid and invalid) and stimulation (Active and Sham) as within‐subject factors, and a 2 × 4 ANOVA with sequence (VV, IV, II, and VI) and stimulation (Active, Sham) as within‐subject factors. Regarding this last analysis and similarly to RT analyses, accuracy related to the second trial of each two‐trial sequence was considered. These analyses were performed to rule out potential speed‐accuracy trade‐offs and to assess whether rTMS over rTPJ affected perceptual discrimination or response caution. The same significance threshold (*p* < 0.05) and reporting criteria (mean ± SEM, with effect sizes expressed as partial eta squared and Cohen's *d*) used for RT analyses were applied.

## 3 Results

**FIGURE 2:**
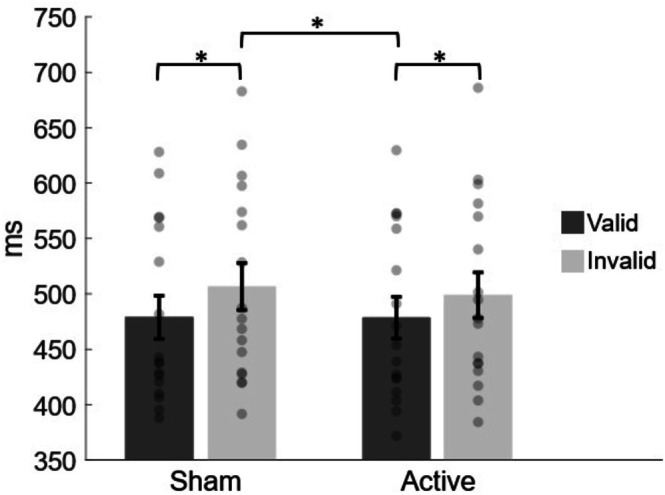
Validity effect results. Mean response times are plotted as a function of both validity and stimulation conditions. Significant effects are shown: the main effect of validity (for both conditions) and the interaction effect between the two conditions due to the significant difference between invalid trials of the Sham condition and valid trials of the Active condition. For each condition, individual data are plotted (gray dots) along with averaged values. Error bars represent variability across participants.

The repeated‐measure ANOVA conducted on mean RTs, considering stimulation and validity as within‐subject factors, revealed a significant main effect of the validity condition (*F*_1,16_ = 39.31; *p* < 0.001; *ƞ* ^2^_p_ = 0.71), highlighting faster RTs in valid (479 ± 19 ms) compared to invalid trials (503 ± 21 ms). The stimulation condition did not reach statistical significance (*F*_1,16_ = 0.50; *p* = 0.49; *ƞ* ^2^_p_ = 0.03), while the interaction between validity and stimulation condition was shown to be statistically significant (*F*_1,16_ = 5.21; *p* = 0.037; *ƞ* ^2^_p_ = 0.245) (Figure 2). Bonferroni post hoc comparisons firstly confirmed the validity effect in both Active (*t*_1,16_ = 5.70; *p* < 0.001, Cohen's *d* = 0.246) and Sham (*t*_1,16_ = 5.91; *p* < 0.001, Cohen's *d* = 0.336) condition. Importantly, only a significant difference between RTs of invalid trials in the Sham condition and RTs elicited by valid trials of the Active condition was observed as well (*t*_1,16_ = 5.29; *p* < 0.001, Cohen's *d* = 0.340) (Figure 2). Therefore, it is acceptable to say that these findings suggest an intact Validity Effect in both Active and Sham stimulations.

**FIGURE 3:**
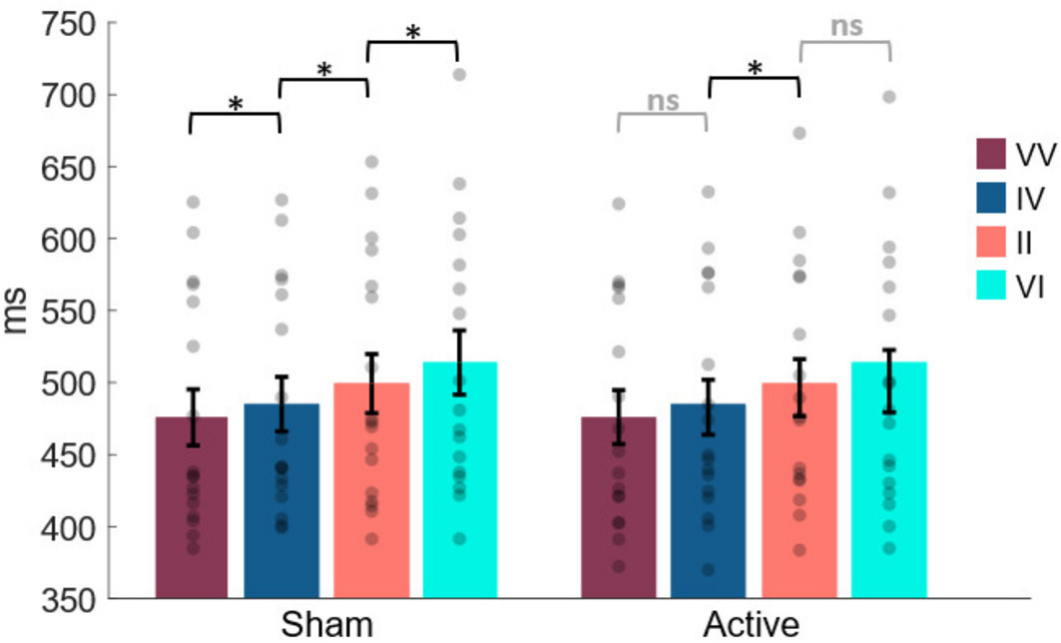
Intertrial validity effect results. Mean response times are plotted as a function of both sequence and stimulation conditions. Significant and not significant effects are shown, these last via gray lines (ns: not significant). For each condition, individual data are plotted (gray dots) along with averaged values. Error bars represent variability across participants.

As to the repeated‐measure ANOVA that took into account stimulation and sequence conditions as within‐subject factors, executed on mean RTs related to the second trial of each two‐trial sequence, it indicated a significant main effect of sequence (*F*_1,16_ = 27.28; *p* < 0.001; *ƞ* ^2^_p_ = 0.63) highlighting the fastest RTs of the second trial for the VV sequence (480 ± 18.8) and the slowest ones for the VI sequence (484 ± 18.7). In contrast, no significant difference in stimulation conditions was found (*F*_1,16_ = 0.57; *p* = 0.46; *ƞ* ^2^_p_ = 0.034). Notably, the interaction between stimulation and sequence reached statistical significance (*F*_1,16_ = 3.97; *p* = 0.013; *ƞ* ^2^_p_ = 0.199). Hence, to further investigate this effect, Bonferroni‐corrected post hoc *t*‐tests were carried out, revealing a crucial difference between Active and Sham conditions, as the following: RTs of the first two sequences, VV (Sham: 476 ± 19.5 ms; Active: 476 ± 18.8 ms) and IV (Sham: 485 ± 18.9 ms; Active: 483 ± 19.1 ms), showed to be significantly different in the Sham (*t*_1,16_ = −5.14; *p* = 0.003, Cohen's *d* = −0.111), while their difference did not reach statistical significance in the Active condition (*t*_1,16_ = −2.92; *p* = 0.28, Cohen's *d* = 0.083). Crucially, the same pattern was unveiled for the last two sequences, II (Sham: 499 ± 20.5 ms; Active: 496 ± 19.8 ms) and VI (Sham: 514 ± 22.3 ms; Active: 501 ± 21.7 ms), which turned out to be significantly different in the Sham (*t*_1,16_ = 3.799; *p* = 0.044, Cohen's *d* = 0.176), but not in the Active condition (*t*_1,16_ = 1.09; *p* = 1.00, Cohen's *d* = 0.054) (Figure 3).

Overall, these results suggest an intact attentional orienting benefit in target discrimination in valid trials, along with an intact cost of rearranging attentional resources in invalid trials, suggesting that rTPJ contribution in reorienting attention toward unexpected locations is not fully tenable. By contrast, the Active TMS stimulation seems to have impacted the intertrial effect, severely reducing the difference between VV and IV and between II and VI. This evidence represents a key behavioral outcome linked to the attentional context updating on a trial‐by‐trial basis.

Furthermore, the 2 × 2 repeated‐measures ANOVA on accuracy with validity and stimulation as within‐subject factors revealed a significant main effect of validity (*F*_1,16_ = 16.484; *p* < 0.001; *ƞ* ^2^_p_ = 0.507), with higher accuracy for valid (96 ± 0.92) compared to invalid trials (94 ± 1.03). Neither the main effect of stimulation (*F*_1,16_ = 0.498; *p* = 0.491; *ƞ* ^2^_p_ = 0.030) nor the interaction between stimulation and validity reached significance (*F*_1,16_ = 0.726; *p* = 0.407; *ƞ* ^2^_p_ = 0.043), indicating that rTMS did not affect perceptual discrimination performance.

Eventually, the repeated measures ANOVA examining accuracy across sequences revealed a significant main effect of sequence (*F*_1,16_ = 29.929; *p* = 0.006; *ƞ* ^2^_p_ = 0.229), with VV yielding higher accuracy than II (*t*_1,16_ = 4.284; *p* = 0.003; Cohen's *d* = 0.430) and VI (*t*_1,16_ = 3.046; *p* = 0.046; Cohen's *d* = 0.470) in both stimulation condition (VV: Active = 96 ± 1.04, Sham = 97 ± 0.8; II: Active = 95 ± 0.99, Sham = 94 ± 1.32; VI: Active = 95 ± 1.08, Sham = 94 ± 1.02), reflecting expected differences in task difficulty across trial patterns. Importantly, no significant main effect of stimulation (*F*_1,16_ = 0.151; *p* = 0.702; *ƞ* ^2^_p_ = 0.009) and no interaction effects were found (*F*_1,16_ = 0.891; *p* = 0.452; *ƞ* ^2^_p_ = 0.053).

Together, these findings confirm that accuracy was consistently maintained at a high level across all conditions and was not modulated by rTMS, ruling out speed‐accuracy trade‐offs and supporting the conclusion that the effects observed in RTs reflect differences in postperceptual, trial‐by‐trial contextual updating rather than changes in perceptual sensitivity or response strategy.

## 4 Discussion

The goal of the present study was to clarify the involvement of rTPJ in visuospatial attentional processes by combining behavioral measures and online rTMS. Refining the knowledge around rTPJ functioning constitutes a remarkable matter due to its widely known implication in a considerable number of cognitive processes and the ensuing cognitive and neuropsychological disorders.

A large number of fMRI studies revealed stronger rTPJ activation in response to invalidly cued targets (i.e., targets appearing in one unattended spatial location that is rather relevant for the behavioral task), accordingly ascribing it to a specific function in triggering attentional reorienting toward unexpected locations (Doricchi et al. 2010; Indovina and MacAluso 2007; Natale et al. 2010; Vossel et al. 2006). However, more recently, it has been argued that this model could not fully account for rTPJ function: More precise tools for identifying the timing of the activation of cortical brain regions have suggested a later rTPJ recruitment (Geng and Vossel 2013; Parisi et al. 2020), which does not match with the reorienting process, which should occur earlier.

Based on these premises, our study has been developed with the purpose of exploiting rTMS's high temporal resolution, along with its likewise good spatial localization power, in order to temporarily interfere with rTPJ activity at predefined timings. Accordingly, we were allowed to observe direct behavioral consequences, uncovered through a location‐cueing paradigm capable of disentangling two essential behavioral effects: the validity effect, representing the implementation of the attentional reorienting process, and the intertrial validity effect, revealing the ability to update one's internal model about the predictive value of the spatial cue.

As to the latter process, TMS interference over rTPJ seems to have an impact on the efficacy of participants' updating the internal representations of cue‐target predictions in a trial‐by‐trial manner. In other words, rTMS affected the interaction between the validity of the previous trial and the validity of the current trial, which rather appears to be unaltered after a Sham stimulation. Going deeper into our intertrial results, Sham data can statistically be described by the following behavioral pattern regarding the RTs linked to the second trial of each two‐trial sequence: VV < IV < II < VI (Arjona and Gómez 2011). Thereby, these results outline the typical circumstances where the reliability conferred to the cue changes with each trial, making the strength in deploying attention to the cued location higher or lower. More precisely, considering the comparison between the first two sequences (i.e., VV < IV), it has been shown that previous valid trials enhanced the reliability assigned to the cue leading attentional resources to be more focused on the direction indicated by the cue during the subsequent valid trial. On the contrary, after an invalid trial, which decreased the reliability of the cue in the following valid trial, the attentional orientation to the target location diminished and caused an increment of RTs for that valid trial. In addition, another crucial piece of evidence concerns the uncovered pattern of II < VI, suggesting that when an invalid trial is preceded by an invalid one, the reliability given to the cue and the attention deployed to the target location are both more attenuated, leading RTs to be faster than in VI trials. Indeed, these last disclosed delayed responses were due to the greater reliability ascribed to the cue, which, however, indicated the wrong direction.

It appears clear that such a bath of processes elicited during this kind of behavioral task depends on the reliability ascribed to the cue, which undergoes a trialwise contextual updating.

The behavioral schemes described above emerge to be influenced by the application of the active TMS, which seems to statistically abolish the significant differences between the first two sequences (i.e., VV < IV) and the last two sequences (i.e., II < VI). The updating process about the conditional cue‐target relationship results in being impacted after rTPJ functioning interference: Without modifying the reliability of the cue, trial‐by‐trial, the benefits or the costs due to the previous trial were reduced, and RTs of the subsequent trials turned out to be comparable for each sequence condition. Additionally, accuracy remained high across all sequence conditions and was not affected by rTMS, ruling out speed‐accuracy trade‐offs and supporting the interpretation that rTMS selectively modulated postperceptual, trial‐by‐trial contextual updating processes rather than perceptual discrimination or response strategies.

Our findings about the functional role of rTPJ in managing the updating of the internal models of a task context align with the existing literature suggesting rTPJ as one of the neural sources of the P3b, which is, in turn, commonly considered a neurophysiological marker of contextual updating.

Of interest, Mengotti et al. (2017) employed online TMS over rTPJ at two predefined timings, namely, 50 or 300 ms after the target onset, showing TMS interference at the late timing only, reducing the participants' ability to update prior beliefs. Our data are further in keeping with a variety of studies (Arjona et al. 2014, 2018; Arjona and Gómez 2013; Arjona Valladares et al. 2017; Gómez et al. 2009, 2019) investigating ERPs components to track the timecourse of events in Central Cue Posner Paradigms, where performance on specific trials highly depends on that of the previous ones. In particular, by grouping trials into two‐trial sequences, Arjona et al. (2014) analyzed P3b data (whose time window perfectly fits our TMS pulse timings) pointing out higher amplitude in the VI sequence than in the II sequence and thus being in line with our results that identify the former sequence as the most impacted one following rTPJ stimulation.

Besides conforming to studies including rTPJ functioning within the contextual updating framework specific to the attentional sphere (Doricchi et al. 2010, 2022; Geng and Vossel 2013; Mengotti et al. 2017; Parisi et al. 2020), our conclusions are additionally corroborated by the fact that the contextual updating theory also provides a reasonable explanation for rTPJ involvement in many other domains, such as the theory of mind and body awareness (Blanke et al. 2005; Decety and Lamm 2007). Indeed, they similarly involve stimulus‐triggered event updating of previous information with new upcoming events provided by the external environment or person or internal body signals (Geng and Vossel 2013; Mengotti et al. 2017) necessary to predict future contingencies related to the specific cognitive process. RTPJ engagement in many cognitive domains can thus be explained by the idea that it plays a global functional role in updating different task‐related contexts depending on its functional connection with those brain networks that come out to be activated during specific cognitive demands (Mengotti et al. 2022; Schuwerk et al. 2021), that is in keeping with the “Overarching view” (Cabeza et al. 2012; Schuwerk et al. 2017, 2021).

Regarding our findings about the validity effect, namely, the attentional benefit deriving from valid trials, together with the attentional cost in responding to unattended targets, depicted by slower RTs in invalid trials, it emerged to be unaltered by rTMS. Comparable validity effects have indeed been found between the rTMS and the Sham conditions, letting us conclude that triggering the reorientation of attention toward uncued but behaviorally relevant spatial locations should not be subserved by rTPJ. Moreover, accuracy analysis revealed the expected main effect of validity, with higher accuracy for valid than invalid trials, but not modulated by rTMS, indicating that attentional reorienting and perceptual discrimination performance were preserved under stimulation. Nevertheless, it is important to consider that rTMS was applied starting 250 ms post‐target, corresponding to a later processing stage associated with the P3b component and contextual updating mechanisms. Previous research indicates that the initial reorientation of attention toward unexpected targets typically occurs within the first 100–250 ms post‐target (Vossel et al. 2012; Chica et al. 2011). Consequently, our findings cannot definitively exclude a contribution of rTPJ to early attentional reorienting, as the applied stimulation may have left unaltered the possibly critical time window necessary to sustain this process. In this respect, while the present results highlight a selective involvement of rTPJ in postperceptual updating, future studies employing rTMS at earlier or multiple time points throughout the trial would be crucial to directly probe its potential role in initiating attentional shifts. Such an approach would provide a more comprehensive picture of the temporal dynamics underlying rTPJ contributions to both reorienting and contextual updating.

## 5 Conclusion

In the current study, we applied online rTMS with the intention of investigating the role of rTPJ in visuospatial attentional mechanisms directly. Taken together, our data disclosed that interfering with rTPJ activity from 250 to 350 ms after the target onset throughout the administration of a location‐cueing paradigm selectively affected participants' updating of the predictive value assigned to the spatial cue, trial‐by‐trial, leaving intact the ability to reorient attention toward uncued behaviorally relevant spatial locations.

Future studies could further explore rTPJ contributions to attentional processes using different experimental manipulations, including variations in the timing of stimulation, to more comprehensively assess its involvement in both reorienting and updating mechanisms.

## Author Contributions

**Giorgia Parisi:** conceptualization, data curation, formal analysis, investigation, methodology, software, validation, visualization, writing – original draft. **Chiara Mazzi:** conceptualization, methodology, writing – review and editing. **Elisabetta Colombari:** formal analysis, writing – review and editing. **Sonia Mele:** conceptualization, data curation, formal analysis, investigation, methodology, software, validation, writing – review and editing. **Silvia Savazzi:** conceptualization, funding acquisition, methodology, project administration, resources, supervision, writing – review and editing.

## Conflicts of Interest

The authors declare no conflicts of interest.

## Data Availability

The behavioral dataset is openly available in the OSF repository at 10.17605/OSF.IO/4UN28.
